# Adapting a Dental Anxiety Measure to Encourage Empathy

**DOI:** 10.1155/2023/4909993

**Published:** 2023-05-22

**Authors:** Margaret Tajirian, Benjamin Juarez, Tomas Martinez

**Affiliations:** Pepperdine University, Malibu, CA 90263, USA

## Abstract

**Introduction:**

The aim of this study was to improve upon the Modified Dental Anxiety Scale (MDAS) by developing the empathy-based International-Modified Dental Anxiety Scale (I-MDAS). This new measure was then utilized to compare the dental anxiety of patients cross-culturally. *Methodology*. This study was a descriptive cross-sectional study adapting the MDAS into the I-MDAS by adding the International scale. The study surveyed 465 participants from a dental clinic, SurveySwap, and distributed flyers. Data was collected through Qualtrics through the self-administered I-MDAS and analyzed through the SPSS computer software version 28. Participants were categorized into two subgroups where 41.3% received dental care only within the United States and are termed the domestic population, and 58.7% received dental care outside of the United States and are labeled the nondomestic population. Information about demographics, past negative dental experiences, and current dental anxiety was collected. The conducted analyses utilized an independent sample *t*-test to compare the subgroups' anxiety levels, a bivariate correlation to find the Pearson correlation, a Cronbach's coefficient *α*, and a one way ANOVA test to compare the genders' I-MDAS scores.

**Results:**

There was no significant difference in dental anxiety levels between the domestic population (*M* = 12.73, SD = 5.13) and the nondomestic population (*M* = 12.76, SD = 5.06); *t* (463) = −0.58, *p* = 0.95). The I-MDAS shows evidence of validity and reliability. There was a significant and positive relationship between the International scale items and the MDAS scale items (*r* (463) = 0.60, *p* < 0.001), indicating the criterion validity of the I-MDAS. Content validity was strengthened by expanding the inquired topics in the new measure. The Cronbach's *α* value of 0.85 shows that the I-MDAS is reliable for clinical applications.

**Conclusions:**

The I-MDAS improves upon the MDAS by providing dentists with a tool for encouraging empathy. Dental clinicians across nations can use the I-MDAS to combat the vicious cycle of dental anxiety.

## 1. Introduction

Dental anxiety is described as a cycle between fear/anxiety, avoidance, deterioration of dental state, and feelings of shame and inferiority toward dentists resulting from genetic, psychological, and social factors [1]. Roughly 36% of the population suffers from dental anxiety [2]. Research identifies the epidemiology of dental anxiety as the following: fear of injections or needles [3], fear of numbing and anesthetics, distrust in dentists due to previous adverse experiences, smoking, poor oral hygiene, the high cost of dental visits, bruxism [4], the perceived lack of control, the lack of empathy from dentists to patients, tooth drilling, and the clinical environment [5]. Largely, one study found that 82.6% of the participants were anxious about tooth extraction [6]. Women have also shown higher dental anxiety levels than men [7–9]. As a result of heightened dental anxiety, people fall into the vicious cycle of anxiety-driven avoidance behavior where they visit the dentist less often, leading to worse self-rated oral health and a greater need for dental treatment [10].

Furthermore, there is limited data regarding dental anxiety varying cross-culturally and what can be done to alleviate dental anxiety in ethnically diverse nations, especially the United States. Thus, the United States' demographic diversity provides a prime opportunity to contrast different cultural backgrounds in correlation to dental anxiety. It is important to note that in seeking to compare diverse patients' dental anxiety levels, the training programs for dentists throughout the world should be considered. Different dental programs utilize a variety of approaches in training student dentists across nations, some enforcing greater scientific advances or new models of dental education [11, 12]. This is not to say that certain programs are superior; however, it is critical to highlight such variations exist and translate into patients' experiences.

As stated above, there is much research supporting the epidemiology of dental anxiety. However, it is now a priority and a requirement to break the vicious cycle of dental fear and avoidance patterns [10]. Thus, this study developed the International Modified Dental Anxiety Scale (I-MDAS which improves upon the Modified Dental Anxiety Scale (MDAS) by increasing content validity. This was accomplished by introducing inquiries regarding two key epidemiological elements: the lack of empathy and patients' distrust of dentists [5]. For reference, content validity demonstrates that the items of a test represent the conceptual domain that the measure is designed to cover [13].

Being empathetic toward patients in healthcare has shown more positive patient and doctor relationships, greater satisfaction, and improvement in patient adherence to treatments and perceptions of health outcomes [14]. Yet, findings demonstrated that empathy is difficult to maintain over time, and professionals' communication skills often decline over the course of their medical careers [15, 16]. As a result, training interventions have been implemented to help reinstate empathy among healthcare practitioners [17]. However, these training sessions do not ensure the improvement of patient–doctor relationships [18]. Unlike the empathy training interventions, the I-MDAS measure can be utilized consistently and objectively with each patient to hold dentists accountable for their empathetic communication toward the individuals they treat.

The I-MDAS is an adapted version of the MDAS, which was utilized as the foundation for this study due to its strong reliability and validity. A variety of previous studies have researched the reliability and validity of the MDAS in accordance with different patient populations, with the lowest Cronbach's coefficient *α* of 0.78 from Nepal [19], and the highest being from an Italian population (0.92) [20].

For this study, it was hypothesized that (1) the domestic group would have varying MDAS scores compared with the nondomestic group, possibly due to differences in dental training programs [21], and (2) the International scale and the MDAS would be positively correlated.

## 2. Methodology

### 2.1. Study Design and Procedure

This was a descriptive cross-sectional study. The survey was provided through Qualtrics and participants were gathered from SurveySwap where the survey was posted to US resident and foreign participants, from dental patients of a participating dental clinic in Los Angeles, and by providing a scannable QR code that was randomly distributed to various neighborhoods in Los Angeles by placing flyers in mailboxes. Data collection occurred from April 2021 to February 2022. More specifically, SurveySwap is a website that sends the survey to participants all over the world. In order to align with the consent form and maintain the privacy rights of the participants, the specific locations of the responders will be withheld; however, generally it may be stated that participants were located in England, China, Netherlands, India, Jordan, Saudi Arabia, North and South Africa, and from a variety of states in the United States including New York, Boston, Kentucky, Texas, and California. Any translations were provided through the website. Participants' ethnicity was also inquired.

The inclusion criteria required that participants were 18 years or older and have the capacity to give consent and be willing to complete the requested surveys and share their thoughts and experiences. All participants who met the inclusion criteria also provided consent. The exclusion criteria included patients who were not mentally sound.

The sample size minimum was found with the population adjustment formula for single proportion estimation [22] and followed another study using a 95% confidence level, a precision of 5%, and power of 0.8 with an expected proportion of 22% [23]. This yielded a minimum of 300 participants required. This study had 465 participants, which increases precision [22]. A stratified random sampling method was utilized and divided the participants into two subgroups: a domestic population (*n* = 192) and an nondomestic population (*n* = 273). The domestic population is defined as participants who have solely experienced dental care within the United States, and the nondomestic population is defined as any participant who has experienced dental care outside of the United States.

### 2.2. Measurements

This study adapted the MDAS [24], which is provided to the public, online. The MDAS is composed of five items for which participants respond on a scale from 1 (not anxious) to 5 (extremely anxious). The total score ranges between 5 and 25. Participants who score above 19 have extreme levels of dental anxiety. The five items measure anxiety in the context of having treatment tomorrow, being in the waiting room, having a tooth drilled, having teeth scaled and polished, and having a local anesthetic injection. Importantly, the MDAS was selected because it has very strong reliability and validity across numerous studies, and it requires a minimal amount of time for completion.

With the addition of five new empathy-based questions, the MDAS was adapted into the I-MDAS (Table S1). The I-MDAS contains two scales: the International scale and the original MDAS scale; together, these comprise the ten items of the I-MDAS. The I-MDAS was created as an empathy-based survey because, while dentists are not expected to be therapists, research has shown that discussing one's challenges and stressors helps to improve their anxiety [25]. Thus, the I-MDAS asks empathy-based items which dentists can utilize to listen, inquire, understand, and connect with the emotions of their patients to help reduce their dental anxiety.

The I-MDAS also has an adapted scoring system according to its two scales (Table S1). The International scale is made up of items one through five. The MDAS scale is made up of items six through ten. Overall, “yes” responses are 1 point and “no” responses are 0 points. Following the numerical scoring of the original MDAS, the possible scores for the I-MDAS were scaled accordingly and are between 7 and 38 [26]. Additionally, the MDAS indicates severe dental anxiety for any score of 19 or above; therefore, the I-MDAS score of 28 out of 38 will indicate severe dental anxiety.

### 2.3. Data Analysis

The data collected was analyzed by using IBM SPSS Statistics (version 28) [27]. To compare the anxiety levels of the domestic and nondomestic populations, an independent sample *t*-test was used. This *t*-test demonstrated the level of significance between the MDAS mean scores of the domestic and nondomestic groups. Normality was measured by testing the skewness of the data. A bivariate correlation was used to find the Pearson correlation to show the relationship between the original MDAS and the International scale and was utilized to indicate the criterion validity of the measure. Reliability analysis for the MDAS and the overall I-MDAS was completed by using Cronbach's coefficient *α*. A one way ANOVA test was utilized to compare the I-MDAS scores of the gender categories.

Ethical clearance was sought from the Pepperdine University Institution Review Board, and permission to conduct the study was obtained from this IRB and the participating dental clinic. Only participants who provided consent were included in this study and all information was handled confidentially. Any objection to participating in this study did not result in negative consequences toward the participant.

## 3. Results

Table 1 illustrates the demographic data of the sample population.

In regard to the first hypothesis, which states that the nondomestic population would have varying MDAS scores compared with the domestic population, a *t*-test showed that the mean MDAS score for the domestic population (*M* = 12.73, SD = 5.13) was not significantly different from the nondomestic population the (*M* = 12.76, SD = 5.06); *t* (463) = −0.58, *p* = 0.95). The MDAS scores are normally distributed as indicated by the measured skewness which was found to be 0.53, falling between 1 and −1.

**Figure 1:**
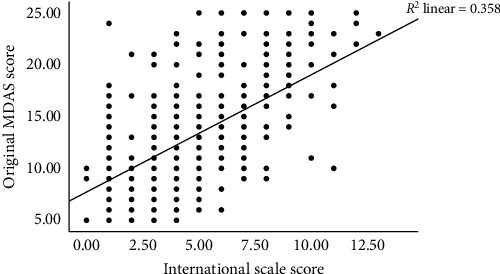
Scatterplot illustrating the significant and positive correlation between the new items of the I-MDAS and the original MDAS items.

To test the second hypothesis, which states that the International scale and the MDAS would be positively correlated, a correlational analysis was used and produced a Pearson coefficient of *r* (463) = 0.60, *p* < 0.001. Thus, as Figure 1 depicts, the new items of the I-MDAS have a significant and positive correlation with the items of the original MDAS. This correlation indicates the criterion validity of the I-MDAS.

The Cronbach's coefficient *α* for the I-MDAS in this study was 0.85 indicating that this new measure is reliable. The Cronbach's *α* coefficient for the MDAS scale alone in this study was 0.88.  Table 2 presents the reliability analysis and item-total statistics, which shows the strength of each item in reference to the Cronbach's coefficient *α* and reliability of the I-MDAS. The deletion of any items has a negligible effect on the Cronbach's coefficient *α*.

Additionally, it was found that there was a significant difference (*F* = 9.65, *p* < 0.001, *η*^2^ = 0.04) between the I-MDAS scores among men (*M* = 15.37, SD = 6.44), women (*M* = 18.29, SD = 7.19), and nonbinary (*M* = 17.8, SD = 4.92), with women producing significantly higher I-MDAS scores on average than men (*p* < 0.001).

Lastly, Table 3 includes personal narratives collected from the optional item 5b (Table S1).

## 4. Discussion

The aim of this study was to improve upon the MDAS by adding items related to empathy. This resulted in the I-MDAS. Past research has focused on the epidemiological elements that contribute to dental anxiety [3–5]. However, this study improves upon the MDAS by adding new items that open effective conversations centered around empathy in dental care. Empathy toward patients has demonstrated improvement in patient cooperation and healthcare experiences [14]. Experiencing meaningful discussions regarding concerns has also demonstrated improvement in anxiety [25]. Thus, the I-MDAS's proactivity will achieve stronger patient–doctor relationships and it will combat the vicious cycle of dental-anxiety-driven avoidance behavior [10].

The first hypothesis, stating that the domestic population would have varying MDAS scores compared with the nondomestic population due to differences in dental training programs across the globe [21], was rejected. This result was due to the insignificant difference in the means of the MDAS scores between the domestic and nondomestic populations. Although the hypothesis was rejected, this result still emphasizes the importance of dentists' awareness toward their patients' backgrounds, as dental history from any country may be pivotal to their level of stress during an appointment [5].

The second hypothesis, stating that the International scale and the MDAS would be positively correlated, was supported as the MDAS and the International scale showed a significant, positive correlation. Thus, the scales' items function together successfully.

Beyond the two hypotheses, the following analyses provided evidence of reliability and validity for the new I-MDAS measure. The I-MDAS increases and strengthens the content validity in which it expands the topics inquired in the original MDAS: it adds items one through five which increase a dentist's awareness of past experiences which may contribute to their patient's anxiety. Additionally, the significant and positive correlation between the MDAS and the International scale supports the criterion validity of this new measure. The I-MDAS is also valuable in that it increased validity, in comparison to the original MDAS, with the addition of new items while there was a negligible impact on the reliability of the original MDAS as shown by the Cronbach's *α* scores. In fact, the MDAS score from this study strongly aligns with the findings of other MDAS reliability studies [19, 20]. Overall, the adapted measure itself produced highly reliable scores.

Furthermore, the I-MDAS was developed to provide an answer to the anxiety-inducing factors which are the lack of empathy and distrust in dentists [5]. The International scale items of the I-MDAS, or the first five questions (Table S1), accomplish this by inquiring about retrospective information that opens a safe space for patients to express their concerns and for dentists to connect with them, utilizing such information to reduce their patient's dental anxiety. Over time, this empathetic communication from dentists or hygienists will likely improve patients' attendance at dental appointments and better their perception of the dental clinic setting [13].

Another finding was that the I-MDAS scores were significantly higher for the women participants of this study compared with the men and nonbinary groups. This finding aligns with previous results where women showed higher MDAS scores compared with men [7, 8, 28] and may be explained by females reporting lower pain tolerance [9]. This is critical in demonstrating how the I-MDAS aligns with the MDAS, not only in its psychometric properties but also in its evidence for producing accurate anxiety level scores in correlation to gender.

Table 3 also displays qualitative results from the participants. This table demonstrates the authentic narratives of patients who share a variety of dental experiences and carry such accounts to each potential visit. These qualitative responses are critical in illustrating the depth of emotional and psychological perceptions that quantitative results do not directly express.

This study experienced limitations. Due to the use of online platforms and a participating dental clinic, a wide variety and a large number of participants were reached; however, these participants did center around the 18–34-year-old population with direct access to the SurveySwap website and the internet. This study also included a larger number of White, female participants. Thus, for future research, it would be beneficial to branch out to other patient populations which could address older age groups, greater variety in sexual orientation, and other ethnicities beyond the limits of Southern California and SurveySwap.

## 5. Conclusion

The findings of this study show that the I-MDAS is an improved measure that has been successfully validated and is reliable for this subject population. The Cronbach's coefficient *α* values show that the I-MDAS is also reliable for clinical applications. The significant and positive correlation between the International scale and the MDAS scale displayed strong validity for their functionality and the overall I-MDAS measure. Importantly, this new measure provides evidence of criterion validity and also increases content validity with a negligible impact on the reliability of the original MDAS. This showcases the strength of this new measure.

These findings encourage dentists across nations to implement the I-MDAS in dental clinics around the world in order to give dentists a quick, accessible tool to further understand each of their patients. It is a guide for the dentist or dental hygienist to perform case-sensitive, empathetic, and individualized treatments to prevent further trauma and promote trust. Future research will include testing the I-MDAS's reliability and validity with other patient populations, as well as further testing for gender differences in I-MDAS scores. This will also allow for test–retest reliability analysis in reference to the future patient populations.

## Figures and Tables

**Table 1:** Demographics of the total study population.

	Total sample (*N* = 465)
Gender (%)	
Men	37.0
Women	61.9
Nonbinary	1.1
Age (%)	
18–24	60.4
25–34	25.6
35–44	5.6
45–54	5.2
55–64	2.6
65–74	0.4
75+	0.2
Ethnicity (%)	
White	68.4
Hispanic or Latinx	6.0
Black or African American	2.4
Native American or American Indian	0.2
Asian American/Pacific Islander	14.2
Other	8.8
Have received dental care (%)	
Yes	95.1
No	4.9
Domestic or nondomestic population (%)	
Domestic	41.3
Nondomestic	58.7

**Table 2:** Results of item-total statistics for the I-MDAS (*N* = 465).

	Scale mean if item deleted	Scale variance if item deleted	Corrected item-total correlation	Cronbach's *α* if item deleted
1. Have you ever received any form of dental care?	17.43	43.56	0.08	0.86
2. Have you had any perceived difficult or adverse experiences with previous dental treatments within the country?	18.08	42.77	0.16	0.86
3. Have you ever had any perceived difficult or adverse experiences with previous dental treatments outside the country?	17.95	42.25	0.22	0.86
4. How do you feel about attending a current dental visit based on your past negative experience?	16.04	32.59	0.74	0.82
5. If you were told a story of someone else's difficult dental visit, how would you feel at a dental appointment?	17.64	42.49	0.21	0.86
6. If you went to your dentist for TREATMENT TOMORROW, how would you feel?	15.91	30.32	0.83	0.81
7. If you were sitting in the WAITING ROOM (waiting for treatment), how would you feel?	15.87	31.13	0.79	0.81
8. If you were about to have a TOOTH DRILLED, how would you feel?	15.04	31.65	0.75	0.82
9. If you were about to have your TEETH SCALED AND POLISHED, how would you feel?	16.03	32.21	0.70	0.82
10. If you were about to have a LOCAL ANAESTHETIC INJECTION in your gum, above an upper back tooth, how would you feel?	15.23	32.57	0.66	0.83

**Table 3:** Twenty descriptive narratives from participants in response to optional item 5b.

Narrative number	Personal narrative
1	She made my teeth sensitive for the past 2 years and she was making rude jokes during the appointment. I have not seen a dentist since.
2	When I was younger I'd have to go and they would work on my mouth and I'd have to hold it open with these crazy brace-type things for what seemed like an hour. It was not fun but not terrible by any means.
3	Many doctors made wrong choices and 8 years later still dealing with the consequences.
4	The doctor was not patient. Didn't let the anesthesia fully set in.
5	I had very bad experiences in the US as a young teen with my dentist who seemingly didn't care about my pain.
6	Nothing medical, just anxiety and sensibility to mild pain during the procedure.
7	After not being able to afford dental care, I had quite a few small cavities and my dentist here in the US was quite judgmental about it. My dentist also misjudged how much anesthesia I would need when filling one of my cavities and that was painful; I dislike going to the dentist now.
8	Not strongly negative, but I've had at least five dentists be incredibly disparaging about my teeth, and refuse to keep chatting with me after they had seen them. This made me extra self-conscious, and less likely to visit as frequently.
9	I chipped an adult tooth close to the nerve as a child and had a bad experience while getting it fixed.
10	Hard to get an appointment, expensive, painful.
11	Over charged, pulled the wrong tooth, took out a piece of my gum…
12	Dentist seems burnt out, not kind or understanding, not even with children.
13	My dentist was horrible, rude, unprofessional, scratched me on accident, and was condescending, also gave wrong advice.
14	I am, like many others, quite scared of the dentist. I don't like examination (always scared they might find something) and I don't like the procedure. Although I can see they do their best and make it as nice as possible.
15	Using the very old method of cleaning teeth with lots of pain.
16	Not the actual treatment per se, but judgment on my dental care from my dentist.
17	Pain, discomfort, dentist constantly leaning against my hair and pulling it.
18	Dentist ignored my request for antibiotics when I claimed to have an infection after a dental operation. Eventually prescribed antibiotics, but had to deal with intense pain for a day first.
19	The local anesthetic often did not fully work or wore off very fast. Some dentists did not take that into account and some even flat out did not believe me when I said I could feel pain.
20	The doctor was unfriendly and constantly joking about me and my teeth.

## Data Availability

The data supporting the conclusions of this study are provided in the results section of this manuscript. Raw data may be requested from the authors; however, it is not provided publicly in order to maintain the confidentiality and privacy of the participants and patients in terms of the consent form contract.

## Supplementary Materials

Supplementary Materials Table S1: International-Modified Dental Anxiety Scale.
