# Supplementary material for: Adapting a Dental Anxiety Measure to Encourage Empathy
Source: Int J Dent. 2023 May 22;2023:4909993. doi: 10.1155/2023/4909993 (PMC10228219; doi:10.1155/2023/4909993)
Supplement: Supplementary Materials — Table S1: International-Modified Dental Anxiety Scale. [file 4909993.f1.docx]

**International Modified Dental Anxiety Scale**

| 1. **Have you ever received any form of dental care?** | | |
| --- | --- | --- |
|  | *Yes* ⬜ | *No* ⬜ |

| 1. **Have you had any perceived difficult or adverse experiences with previous dental treatments within the country?** | | |
| --- | --- | --- |
|  | *Yes* ⬜ | *No* ⬜ |

| 1. **Have you had any perceived difficult or adverse experiences with previous dental treatments outside the country?** | | |
| --- | --- | --- |
|  | *Yes* ⬜ | *No* ⬜ |

| 1. **How do you feel about attending a current dental visit based on your past negative dental experience?** | | | | | |
| --- | --- | --- | --- | --- | --- |
|  | *Not*  *Anxious* ⬜ | *Slightly*  *Anxious* ⬜ | *Fairly*  *Anxious* ⬜ | *Very*  *Anxious* ⬜ | *Extremely*  *Anxious* ⬜ |

| 1. **If you were told a story of someone else’s difficult dental visit, how would you feel at a dental appointment?** | | | | | |
| --- | --- | --- | --- | --- | --- |
|  | *Not*  *Anxious* ⬜ | *Slightly*  *Anxious* ⬜ | *Fairly*  *Anxious* ⬜ | *Very*  *Anxious* ⬜ | *Extremely*  *Anxious* ⬜ |

| **Could you explain a past difficult dental experience or story you heard from someone else’s experience?** | |
| --- | --- |
|  |  |

| 1. **If you went to your Dentist for TREATMENT TOMORROW, how would you feel?** | | | | | |
| --- | --- | --- | --- | --- | --- |
|  | *Not*  *Anxious* ⬜ | *Slightly*  *Anxious* ⬜ | *Fairly*  *Anxious* ⬜ | *Very*  *Anxious* ⬜ | *Extremely*  *Anxious* ⬜ |

| 1. **If you were sitting in the WAITING ROOM (waiting for treatment), how would you feel?** | | | | | |
| --- | --- | --- | --- | --- | --- |
|  | *Not*  *Anxious* ⬜ | *Slightly*  *Anxious* ⬜ | *Fairly*  *Anxious* ⬜ | *Very*  *Anxious* ⬜ | *Extremely*  *Anxious* ⬜ |

| 1. **If you were about to have a TOOTH DRILLED, how would you feel?** | | | | | |
| --- | --- | --- | --- | --- | --- |
|  | *Not*  *Anxious* ⬜ | *Slightly*  *Anxious* ⬜ | *Fairly*  *Anxious* ⬜ | *Very*  *Anxious* ⬜ | *Extremely*  *Anxious* ⬜ |

| 1. **If you were about to have your TEETH SCALED AND POLISHED, how would you feel?** | | | | | |
| --- | --- | --- | --- | --- | --- |
|  | *Not*  *Anxious* ⬜ | *Slightly*  *Anxious* ⬜ | *Fairly*  *Anxious* ⬜ | *Very*  *Anxious* ⬜ | *Extremely*  *Anxious* ⬜ |

| 1. **If you were about to have a LOCAL ANAESTHETIC INJECTION in your gum, above an upper back tooth, how would you feel?** | | | | | |
| --- | --- | --- | --- | --- | --- |
|  | *Not*  *Anxious* ⬜ | *Slightly*  *Anxious* ⬜ | *Fairly*  *Anxious* ⬜ | *Very*  *Anxious* ⬜ | *Extremely*  *Anxious* ⬜ |

***The International Modified Dental Anxiety Scale.* Each item scored as follows:**

Yes = 1

No = 0

Not anxious = 1

Slightly anxious = 2

Fairly anxious = 3

Very anxious = 4

Extremely anxious = 5

**MDAS Score: Q6 + Q7 + Q8 + Q9 + Q10**

**International Scale Score: Q1 + Q2+ Q3 + Q4 + Q5**

**MDAS Score + International Scale Score = I-MDAS Total Score: ______**
